# The relationship between paternal education, self-esteem, resilience, future orientation, and career aspirations

**DOI:** 10.1371/journal.pone.0243283

**Published:** 2020-12-08

**Authors:** Buratin Khampirat

**Affiliations:** Suranaree University of Technology, Institute of Social Technology, Nakhon Ratchasima, Thailand; University of Sao Paulo Medical School, BRAZIL

## Abstract

Career aspirations are considered to be one of the most important motivation variables in the study of psychology and career development, as well as being connected to an individual’s career-related goals, intentions or options. The aims of this study were: (a) to develop and validate a model for predicting career aspirations, and (b) to investigate direct and indirect links between paternal education, self-esteem, resilience, future orientation, and career aspirations of university students. The participants were 445 students from two universities in Thailand. Confirmatory factor analysis confirmed that the factor structure of four measurement models presented in the study was suitable and compatible. To achieve the intended research objectives, the empirical data were analyzed using structural equation modeling with decomposition analysis of total effects in direct and indirect effects. Results showed that paternal education, resilience, and future orientation have significant direct effects on students’ career aspirations, while self-esteem has an indirect effect. This suggests that self-esteem can help students develop their resilience, as well as promoting their development of a positive future orientation, which also helps foster a higher level of career aspiration. These results can be fundamental to supporting the continued use of the structural equation model in future research on career development.

## Introduction

In the current competitive labor market, it is increasingly challenging for higher education institutions to produce graduates that are well prepared for employment [1]. One of the most effective approaches to preparing students for employment is to create and develop career aspirations and career goals which motivate and inspire those students with regard to their employability and future careers [2, 3]. Career aspirations are defined as the desire and intention to pursue occupational goals through particular career development processes [4, 5], can also encompass the benefits and significant determinants of both short-and long-term career goals and future career mobility [6].

Career aspirations are often linked to the political agendas of some developed and developing countries, with social mobility and the enhancement of work skills forming a basis for economic growth [7, 8]. In general, students’ career aspirations are driven by their status in society [9–13]. Previous research has highlighted the notion that demographics, family characteristics, social class, and socioeconomic status affect career aspirations [4, 14–16], which are important to improving the achievement of children and adolescents [17].

A study on high school students revealed that parental educational level contributed to students’ educational and occupational aspirations and parents with prestigious professions tended to encourage their children to aspire to their professions [18]. It appeared that parents from higher socioeconomic status (with prestigious professions and/or high educational level) have higher educational and career expectations for their children compared with parents from lower socioeconomic status [19]. Another study showed positive relationships between parental education level and parents’ expectations for the success of their children [20]. Khallad [21], Mau and Bikos [22], and Watson, Quatman [11] reported that career aspirations could be affected by parental education and socioeconomic status, encompassing the opportunity, encouragement, and support that parents provide to children [23], in terms of their aspirations and career development.

In the past decades, a number of research studies have been conducted on the relationship between self-esteem and career aspirations [24–29], the results of which demonstrated that having high self-esteem would encourage students to maintain a positive outlook on career aspirations related to their present or future performance after graduation. Studies have shown that the role of career development is to enforce one to achieve a future orientation, which is described by “the ability to planfully look ahead” [30, 31]. Because resilience can affect the ways in which children perceive themselves and can help improve their aspirations to achieve the goal of education [32], resilience was hypothesized to be one of the mediators in this study; as socio ecological resources that can counterpose the negative development effects [32], resilience was suggested to be connected to the efforts to succeed in education [33]. A study suggested that resilience can be strengthened through counseling processes, career courses, and workshops [34].

In the context of the Thai higher education, factors related to resilience in undergraduate nursing students were studied using descriptive statistics [35]. The results showed that half (50.50%) of the students perceived themselves as having higher resilience than an average and most of them (73.80%) perceived their family atmospheres as good. Other variables such as adversity quotient and relationships with friends were also shown to be significantly associated with resilience. A review of the literature shows that there are many factors which may affect the career aspirations of students. These factors can also guide students and those involved in designing and planning students’ career development, and support the achievement of their goals in future careers.

In Thailand, as in other developing countries in general nowadays, most students from lower-income families are encouraged to attain higher levels of education than did their parents [36]. Because of the Thai cultural context, family environment and parents tend to be a crucial factor in shaping students’ career choices [10, 37]. This is reflected in the trends in the career decision-making of Thai students, in which the family cultural system is very influential. Studies show that students from rural areas and from low-income families are more often attracted to low skill careers [10].

Students’ attitude toward career achievements should clearly be addressed within the context of their specific culture [38], but career aspirations from the perspective of university students are not being widely studied in Thailand. Therefore, the aims of this study were: (a) to develop and validate a model for predicting career aspirations, and (b) to investigate direct and indirect links between paternal education, self-esteem, resilience, future orientation, and career aspirations of university students in Thailand. The information obtained in this research study is expected to assist career counselors, lecturers, policy makers and parents to develop students’ career aspirations, and thus enable them to achieve their professional goals.

### Understanding career aspirations

The term “aspiration” is widely used in reflecting the future lifestyle, dreams, desires and ambitions of individuals [8]. Studies have shown that raising aspirations can lead to higher achievement, both in careers and education [8, 39]. The concepts of aspiration applied in this study are rooted in the theory of achievement motivation [40], and the theory of social comparison processes [41], which are related to person's career aspirations. In the study of psychology and career development, career aspirations are considered to be one of the most important motivation variables [42, 43], and are connected to an individual’s career-related goals, intentions or options [4, 44]. These aspirations are influenced by self-determination, according to the self-determination theory [45], which focuses on fundamental issues to address the importance of three psychological needs (e.g., competence, autonomy, and psychological relatedness) [45] for an individual’s well-being, satisfaction, and optimal performance [46]. Aspects of career aspirations include an orientation toward future educational and occupational goals which are associated with an individual’s future life [4, 22]. Furthermore, there are several other variables associated with career aspirations, e.g., age, gender, family background, socioeconomic status, social capital, school attainment, self-esteem, self-efficacy [14, 29]. It has been stated that the learning environment in the classroom and on campus also has a strong relationship with individual aspirations and satisfaction with the university. A meta-analysis carried out by Akosah-Twumasi, Emeto [47] and a study conducted by Fouad, Kim [48] emphasize that a complex interaction of cultural and environmental contexts affects the career aspirations of youths. Moreover, Gonzalez, Stein [49] suggest that students from immigrant families who feel a positive ethnic experience at school will have more confidence in their career aspirations.

In addition, lecturers and policymakers can help improve the learning environment and increase students’ aspirations through fostering “the-love-of-learning” [50]. Castro and Armitage-Chan [26] investigated the relationship between gender, self-esteem, year of study and career aspirations of veterinary students in the United Kingdom. Their study indicated that self-esteem, year of study, confidence, and previously holding a position in the students’ society were significant predictors of career aspirations. Conversely, based on the analysis of data obtained in a longitudinal study conducted by Rojewski and Yang [51], it was found that in general, self-esteem had little effect on the career aspirations of American adolescents.

Interestingly, the results of several studies indicated that rural students possessed lower educational and career aspirations than their urban peers [40, 52, 53]. On the other hand, other studies have shown no solid evidence indicating that students from poor family backgrounds or the more disadvantaged social classes have higher aspirations than other students [54–56].

Although several interesting influences on career aspirations have been identified, to better understand students’ career development, further studies regarding their quality of life and future employment are still necessary.

### Conceptual framework and hypotheses

**Fig 1:**
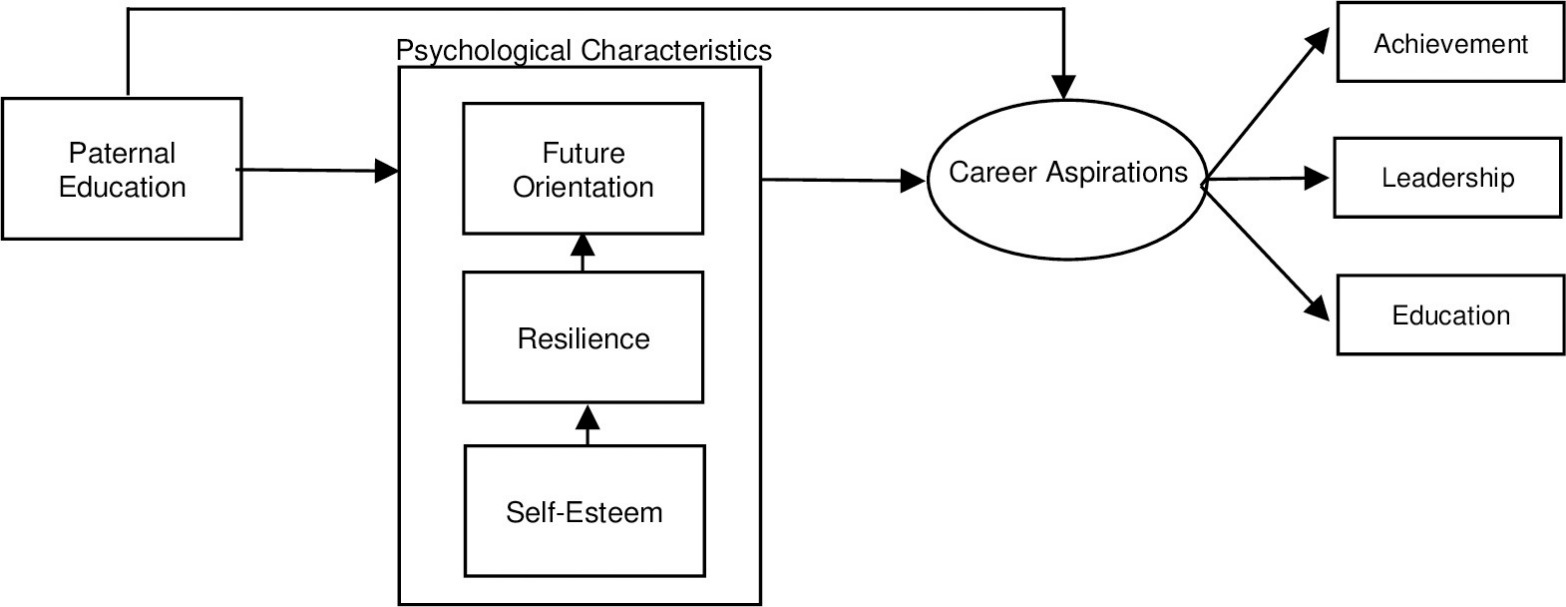
Conceptual framework.

Fig 1 illustrates the conceptual framework of the study and identifies the relationship between constructs in the structural model. Based on the concepts of career aspirations, theories in the field, and previous empirical studies, the conceptual framework developed in this study focused on the relationships between paternal education level, students’ psychological characteristics (such as self-esteem, resilience, and future orientation), and career aspirations as an outcome variable. Three hypotheses were determined for the study as follows:

Hypothesis 1: Paternal education would have a positive significant related to self-esteem, resilience, and future orientation.

Hypothesis 2: Self-esteem, future orientation, and resilience would have significant positive direct and indirect effects on career aspirations.

Hypothesis 3: Paternal education level would have significant positive results, both direct and indirect, in predicting career aspirations.

## Materials and methods

### Participants

The participants in this study were 445 undergraduate students (response rate = 91.75%) in two universities (of technology), one of which was a prestigious public autonomous research university (one of the top nine well-funded research universities in Thailand) with strong science and engineering programs, and the other was a vocational university. The former is located in a populated city in the lower north-eastern region, and the latter is in the eastern industrial seaboard area of Thailand. Participants were recruited using a convenience sampling method during September 2017–March 2018. They were undergraduate students aged between 17 and 26 years. The inclusion criteria were the students, who were willing to participate in the survey. The exclusion criteria were students who were not convenient or comfortable to participate in the study and incomplete returned questionnaires with missing responses more than 10% for a particular variable. Because the participants were either freshmen or senior, the study sample of 445 can be representative of large population (>100,000) at the 95% confidence level [57, 58], especially in terms of gender and parents’ education.

Demographic characteristics of participants are included in Table 1. The majority of participants (n = 289, 71.17%) were female, and 156 (35.06%) were male. The field of study of these participants was diverse; 216 of them studied information technology (48.58%), 132 studied management (29.66%), 34 studied agricultural technology (7.64%), and 63 were students from other fields (14.16%). Regarding the highest educational attainment of the their parents, participants stated that: (1) 23.82% of fathers and 25.62% of mothers had attained an elementary education or lower, (2) 34.83% of fathers and 37.30% of mothers had attained secondary education, (3) 12.81% of fathers and 9.66% of mothers had attained vocational and high vocational education, (4) 21.57% of fathers and 21.57% of mothers had attained a Bachelor’s degree, and (5) 4.72% of fathers and 3.14% of mothers had attained a Master’s degree or above.

**Table 1:** Demographic characteristics of participants.

Demographic Variables		Frequency	%
Gender	Male	156	35.06
	Female	289	64.94
	Total	445	100.00
Major	English	31	6.97
	Economics	32	7.19
	Agricultural Business	34	7.64
	Management	132	29.66
	Information Technology	216	48.54
	Total	445	100.00
Year of Study	First Year	216	48.54
	Fourth Year	229	51.46
	Total	445	100.00
Area of High School that Students Graduate From	Rural	37	8.31
	Urban	391	87.87
	Capital	17	3.82
	Total	445	100.00
Father's Education	Less than Secondary School (≥ 6 Years)	106	23.82
	Secondary School (7–12 Years)	155	34.83
	Training for Career-Path or TVET Certificate (13–14 Years)	57	12.81
	Bachelor Degree (16 Years)	96	21.57
	Master’s Degree (18 Years)	19	4.27
	Doctorate Degree (22 Years)	2	0.45
	N/A	10	2.25
	Total	445	100.00
Mother's Education	Less Than Secondary School (≥ 6 Years)	114	25.62
	Secondary School (7–12 Years)	166	37.30
	Training for Career-Path or TVET Certificate (13–14 Years)	43	9.66
	Bachelor Degree (16 Years)	96	21.57
	Master’s Degree (18 Years)	13	2.92
	Doctorate Degree (22 Years)	1	0.22
	N/A	12	2.70
	Total	445	100.00

### Measures

A self-report instrument was developed to measure the variables for this research. The details of all the instruments used in the study are as follows:

#### Career aspirations

In measuring the outcome variable, the study focused on career ambitions, based on the concept proposed by O'Brien [59]. The research further made use of a revised version of the career aspiration scale (CAS) developed by Gregor and O’Brien [60], which consisted of three subscales, including achievement (8 items), leadership (8 items), and educational (8 items). Participants used a Likert-type scale that ranged from 0 (not at all true for me) to 4 (very true for me) to rate their level of aspiration in each area. Scores in each subscale, therefore, ranged from 0 to 32, with higher scores reflecting higher career aspirations [60]. The response of each CAS subscale was summed to obtain its total score; higher scores reflected stronger CAS. The criteria for interpretation the scale’s mean were ≥ 25.60 very well, ≥ 22.40 good, ≥ 19.20 fair, ≥ 16.00 poor, and < 16.00 very poor. Examples of items in the subscales were as follows: in the achievement aspirations subscales, “I want to be among the very best in my field”; in the leadership aspirations subscales, “I hope to become a leader in my career field”; and in educational aspirations subscales, “I plan to reach the highest level of education in my field”. The Cronbach’s alpha coefficients for the three subscales showed an acceptable level of reliability, ranging between 0.81 and 0.92.

#### Self-esteem

To assess how the students felt about themselves, the Rosenberg’s self-esteem scale [61] was used. This is a tool for assessing global self-worth, consisting of 10 self-report items. It was used as an indicator of the observed “self-esteem” variable in this study. The statements were rated along a 4-point Likert-type scale ranging from strongly disagree (1) to strongly agree (4). For response rated from 1 to 4, this study used the following criteria for interpretation of weighted mean; 1–1.75 poor, >1.75–2.5 fair, > 2.5–3.25 good, and > 3.25–4.0 very good [62]. The scale was found to have adequate reliability (α = 0.73).

#### Future orientation and resilience

Di Maggio, Ginevra [63] developed future orientation and resilience scales, based on the perspective of the relevant scholars, to measure future orientation and resilience constructs in adolescents involved in career planning. Eleven items of the future orientation scale and eight items of the resilience scale were adopted in this study, and responses were given on a 5-point scale, ranging from 1 (It describes me not at all) to 5 (It describes me very well). For response rated from 1 to 5, this study used the following criteria for interpretation of weighted mean; 1–1.8 = very poor, > 1.8–2.6 = poor, > 2.6–3.4 = fair, > 3.4–4.2 = good, and > 4.20–5.0 = very well [62]. The Cronbach’s alpha coefficients were 0.86 for future orientation and 0.87 for resilience.

#### Paternal education

In this research, because demographic data analysis in Table 1 showed that on average, the highest educational levels of father and mother are not significantly different, the highest education level of father was used as a predictor variable. This choice was supported by the fact that in the Thai society, fathers play a very strong role as leaders of the family, and are expected to be family breadwinners [64].

In statistical analysis concerning with the measurement of level of education, it is necessary to translate the level of education into the number of years of educations [65]. Especially in this work, the level of education was one of the predictor variables and therefore must be numeric. Paternal education levels were transformed into the number of years of education according to the maximum level of education attained [65] based on the educational system in Thailand; the number of years of educations can be directly correlated to the level of educations e.g., the primary (6 years) and secondary (12 years) school certificates or university Bachelor’s degrees (16 years).

#### Demographic information

The participants were asked to report their gender, type of study program, and experience in the workplace through co-operative education.

### Data analyses

Statistical analyses were conducted using SPSS 18.0 and Mplus 8.3 software [66]. Descriptive statistics were calculated to assess the normality of distribution. The Pearson correlation (*r*) matrix was used to examine the relationship between the variables in the model. Cronbach's Alpha coefficient (α) was assessed to measure the measurement scale reliability. Confirmatory factor analysis (CFA) was carried out to confirm the factor structure of the measurement model. Structural equation modeling (SEM) was conducted to explore causal relationships of the career aspirations structural model.

Assessing the goodness of fit of the model was conducted by using the ratio of χ^2^ to the degrees of freedom (*χ*^*2*^*/df*, acceptable if < 3), the comparative fit index (CFI, acceptable if ≥ 0.90), Tucker-Lewis index (TLI, acceptable if ≥ 0.90), the root mean square error of approximation (RMSEA, acceptable if < 0.06 to 0.08 with 90% confidence intervals), and the standardized root mean squared residual (SRMR, acceptable if ≤ 0.08) [67, 68].

### Procedures

This study was a cross-sectional study conducted in accordance with ethical principles and guidelines for research in human subjects. The ethical approval was granted by Suranaree University of Technology, Thailand (EC-61-93). The Thai versions of all four instruments (CAS, self-esteem, resilience, and future orientation) were developed using back-translation from English into Thai by the researcher and an expert in the English language. The questionnaires in English [60, 61, 69] and Thai were included as (S1 Questionnaires). The anonymous surveys were administered by lecturers in the participants’ classrooms, who were provided with detailed instructions regarding the questionnaire. At the end of the class, all participants were given a copy of the informed consent form, which outlined the title of study, details of the principal investigator, purposes of study, procedures, and confidentiality measures. Students were informed that participation was completely voluntary and students who did not want to participate were free to leave. They were also assured that all records identifying their background and responses will be kept confidential and that the information would be presented only as a whole.

## Results

### Descriptive statistics and intercorrelations among variables

The dataset for investigating the relationship between paternal education, self-esteem, resilience, future orientation, and career aspirations were included as (S1 Dataset). Mean (*M*), standard deviations (*SD*), skewness (*SK*), and kurtosis (*KU*) of the constructs and each item are displayed in Table 2. The mean scores of three subscales of CAS showed that Thai students reported a slightly higher achievement aspiration (*M* = 24.13, *SD* = 4.42) than educational (*M* = 23.91, *SD* = 4.09), and leadership aspirations (*M* = 22.78, *SD* = 5.11). The mean of future orientation (*M* = 2.96, *SD* = 0.51) and resilience (*M* = 3.75, *SD* = 0.57) was in good levels. The same applied to self-esteem, which was in a good range (*M* = 3.00, *SD* = 0.30) on a 4-point scale. Besides, the average number of years for the highest education of fathers was 11.62 years (*SD* = 4.02), which meant that the average level was high school level. *SK* values ranged from -0.16 to -1.43 (*SK* < |3|), and *KU* values ranged from -1.02 to 1.87 (*KU* < |10|) [70], indicating that data was taken from a normally distributed population. Table 3 presents the Pearson correlation coefficient between all variables, ranging from 0.255 to 0.975. All three domains of CAS had a significant positive relationship (*p* < 0.01) to each predictor variable.

**Table 2:** Descriptive statistics.

Variable	Item	N	Min.	Max.	*M*	*SD*	*SK*	*KU*
Achievement Aspiration		445	8.00	32.00	24.13	4.42	-0.37	-0.28
	CAS_3	445	0	4	3.03	0.92	-0.52	-0.69
	CAS_8	445	1	4	3.17	0.84	-0.72	-0.22
	CAS_9	445	0	4	3.10	0.80	-0.55	-0.12
	CAS_13	445	0	4	2.73	0.95	-0.49	-0.02
	CAS_17	445	0	4	2.90	0.83	-0.23	-0.58
	CAS_20*	445	0	4	3.32	0.91	-1.43	1.87
	CAS_21	445	0	4	2.93	0.99	-0.87	0.57
	CAS_22*	445	0	4	2.95	0.99	-0.69	-0.14
Leadership Aspiration		445	4.00	32.00	22.78	5.11	-0.35	-0.52
	CAS_1	445	0	4	2.68	1.06	-0.64	-0.10
	CAS_2*	445	0	4	2.96	0.93	-0.66	0.17
	CAS_4*	445	0	4	2.86	1.04	-0.63	-0.26
	CAS_5	445	0	4	2.38	1.14	-0.25	-0.65
	CAS_7	445	1	4	3.18	0.78	-0.58	-0.35
	CAS_12*	445	0	4	2.71	1.03	-0.39	-0.44
	CAS_15	445	0	4	3.00	0.88	-0.54	-0.29
	CAS_24	445	0	4	3.02	0.89	-0.51	-0.54
Educational Aspiration		445	7.00	32.00	23.91	4.90	-0.25	-0.52
	CAS_6	445	1	4	3.00	0.90	-0.34	-1.02
	CAS_10	445	1	4	3.25	0.74	-0.70	-0.01
	CAS_11	445	0	4	2.89	0.90	-0.47	-0.02
	CAS_14	445	1	4	2.95	0.81	-0.29	-0.62
	CAS_16	445	0	4	2.91	0.87	-0.32	-0.63
	CAS_18	445	0	4	3.03	0.88	-0.54	-0.33
	CAS_19	445	0	4	2.95	0.92	-0.62	0.03
	CAS_23	445	0	4	2.94	0.90	-0.49	-0.23
Future Orientation		445	2.18	5.00	3.96	0.51	-0.16	-0.27
Resilience		445	1.25	5.00	3.75	0.57	-0.19	0.84
Self Esteem		445	1.40	3.80	3.00	0.39	-0.44	0.27
Years of Education of Father		435	0	22	11.62	4.02	-0.23	-0.93

Note. *SE*_*SK*_ = 0.12, *SE*_*KU*_ = 0.23;

* = reverse-scored.

**Table 3:** Alpha and intercorrelations among variables.

Variable		α	1	2	3	4	5	6	7
Career Aspirations	1. Achievement Aspiration	0.76	1.000						
	2. Leadership Aspiration	0.81	.977**	1.000					
	3. Educational Aspiration	0.86	.975**	.967**	1.000				
Predictor Variables	4. Future Orientation	0.86	.760**	.755**	.761**	1.000			
	5. Resilience	0.87	.687**	.690**	.686**	.690**	1.000		
	6. Self-Esteem	0.73	.367**	.349**	.351**	.402**	. 528**	1.000	
	7. Years of Education of Father		.751**	.753**	.750**	.572**	.529**	.255**	1.000

Note. *N* = 445

* *p* < 0.05,

** *p* < 0.01.

### Assessing the measurement model validity

**Fig 2:**
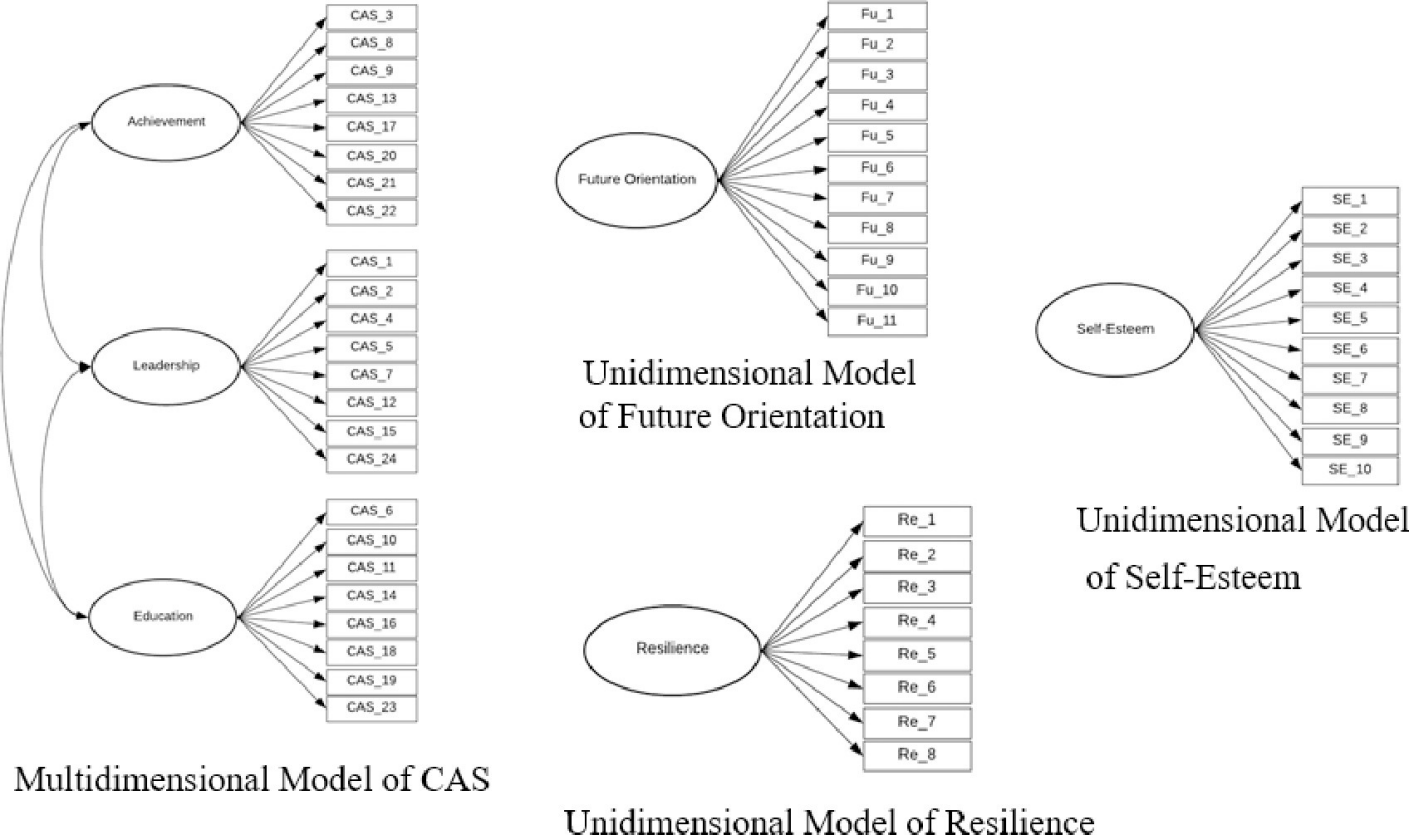
Measurement models tested.

Table 4 displays goodness of fit statistics for the four different measurement models from the CFA output; the multidimensional model of CAS, and unidimensional models of future orientation, resilience, and self-esteem (see Fig 2). The results showed that all the measurement models presented adequate goodness-of-fit indices. However, in this research, SEM was adopted to explore the relationships between variables in the model that required large sample size of at least 20 cases for each estimated parameter in the model [71] to ensure sufficient statistical power for significance tests and overall fit. Therefore, due to the limitations of the sample size in the present study (445 participants), each construct was considered as an observed numerical variable. An exception was the CAS, which was still measured in form of latent variable consisting of three indicators.

**Table 4:** Goodness of fit statistics for the measurement models tested.

Construct	χ^2^	df	χ^2^/df (< 3)	CFI (≥ 0.90)	TLI (≥ 0.90)	SRMR (≤ 0.08)	RMSEA (90% CI) (< 0.06 to 0.08)
1. Career Aspiration (24 Items)	441.98 (*p* < 0.001)	191	2.31	0.95	0.92	0.06	0.062 (0.055–0.070)
2. Future Orientation (11 Items)	67.982 (*p* = 0.001)	37	1.84	0.97	0.96	0.03	0.043 (0.027–0.059)
3. Resilience (8 Items)	22.134 (*p* = 0.076)	14	1.58	0.99	0.99	0.02	0.036 (0.000–0.063)
4. Self-Esteem (10 Items)	77.151 (*p* < 0.001)	26	2.97	0.96	0.93	0.04	0.066 (0.050–0.084)

### Prediction of career aspiration

**Fig 3:**
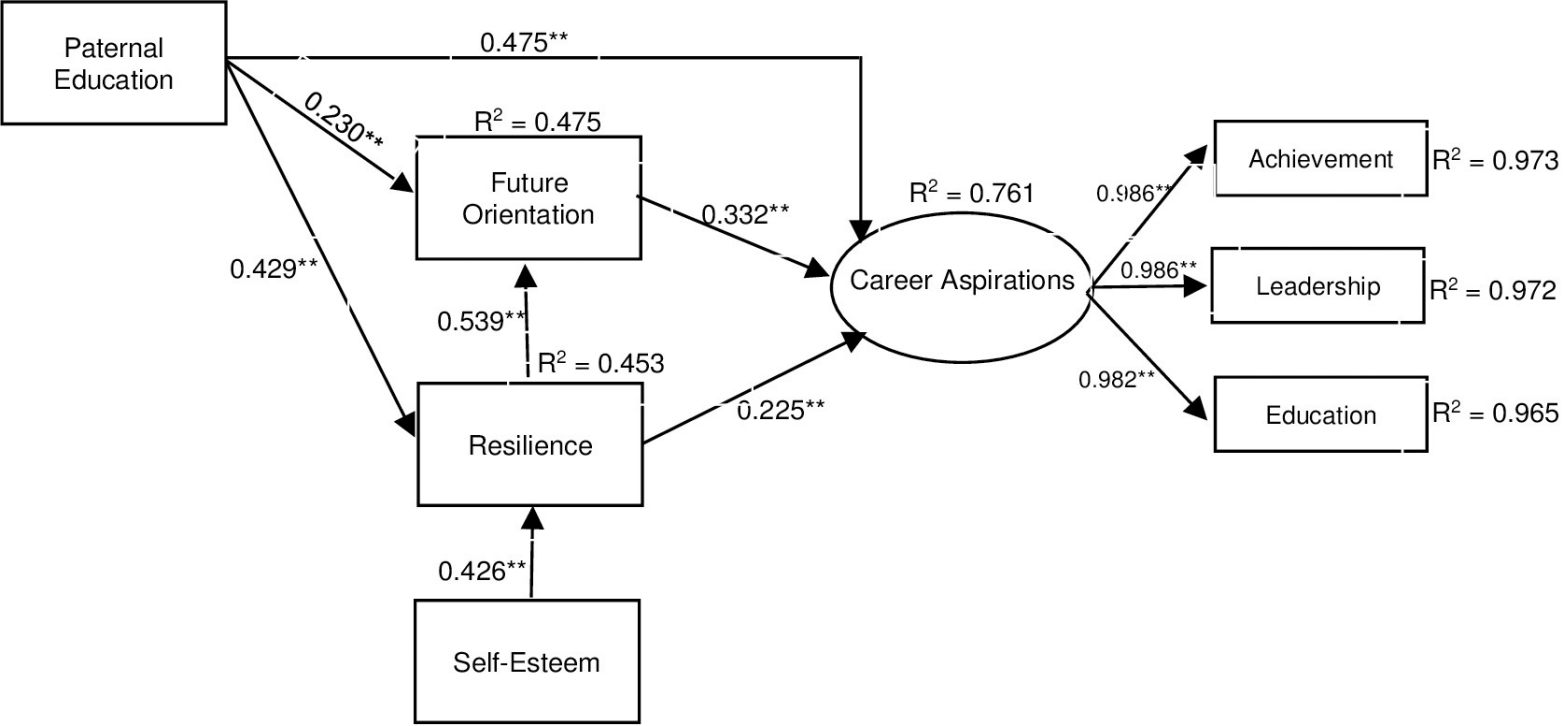
Standardized path coefficients of structural equation modeling for career aspirations. * *p* < 0.05, ** *p* <0.01.

Based on the conceptual framework, SEM was performed to explore the effects of paternal education level, self-esteem, future orientation, and resilience on career aspirations. The resulting career aspiration SEM is presented in Fig 3 and Table 5, showing that the hypothesized model was a good fit with the observed data, *χ*^*2*^(10) = 28.154; *p* = 0.002, *χ*^*2*^*/df* = 2.815, CFI = 0.994, TLI = 0.997, RMSEA = 0.073 (90% confidence interval = -0.042 to 0.106), SRMR = 0.014. All of the standardized path coefficients were statistically significant and had a positive direct effect on career aspirations. The slightly high upper range for the 90% confidence interval of RMSEA (> .08) could be attributed to the sampling uncertainty that might be affected by sample size and the model complexity [72, 73].

**Table 5:** Standardized path coefficients of constructs and their significant values.

Path (effects from X to Y)	Direct Effect	Indirect Effect	Total Effect
Paternal Education ➔ CAS	0.475** (14.621)	0.249 ** (10.658)	0.724** (28.816)
Self-Esteem ➔ CAS	-	0.172** (7.550)	0.172** (7.550)
Resilience ➔ CAS	0.225** (5.929)	0.179** (7.138)	0.404** (11.543)
Future Orientation ➔ CAS	0.332** (8.833)	-	0.332** (8.833)

Note. Values in parentheses are Z-values;

* *p* < 0.05,

** *p* < 0.01.

For the career aspiration measurement model, the results showed that three standardized factor loadings were significant, and higher than 0.50, indicating that the three subscales contributed significantly to the measurement of career aspirations. The SEM results indicated that the hypothesized structural model for career aspirations adequately fitted the data. The set of four predictor variables in the model explained 76.1% (*R*^*2*^ = 0.761) of the variance in career aspirations. Hypothesis 1 was confirmed only for future orientation and resilience, whereas self-esteem was not confirmed. Results indicated that paternal education level had a significant positive relationship with future orientation (*β* = 0.230, *p* < 0.001) and resilience (*β* = 0.429, *p* < 0.001), while there was no relationship between paternal education and self-esteem. Hypothesis 2 was also partially confirmed, as the path coefficients between future orientation (*β* = 0.332, *p* < 0.001) showed a significant positive direct effect on career aspirations, whereas resilience had both a strong positive direct (*β* = 0.225, *p* < 0.001) and indirect (*β* = 0.179, *p* < 0.001) impact on career aspirations, mediated by future orientation. The total effect of resilience was 0.404 (*p* < 0.001). Hypothesis 3 also confirmed that paternal education showed a strong predictive relationship with the other constructs in this model; in predicting the level of career aspirations, paternal education level was significant with regard to positive effects, both direct and indirect. The standardized direct effect of paternal education level on career aspirations was 0.475 (*p* < 0.001), while the total indirect effect was 0.249 (*p* < 0.001). The total effect from paternal education level to career aspirations was 0.724 (*p* < 0.001).

In addition, the results obtained from the SEM indicate that self-esteem has a positive indirect effect on career aspirations through future orientation and resilience (*β* = 0.172, *p* < 0.001). These findings describe the role of resilience and future orientation as mediating variables which aid in understanding the relationship between paternal education and forms of self-esteem, and the career aspirations of Thai university students.

## Discussion

Based on the findings in this study, the conclusion drawn was that paternal education level, self-esteem, resilience, and future orientation can play an important role in the career aspirations of Thai students. The results showed that paternal education level had the largest positive association with the career aspirations of Thai university students. In other words, Thai students whose fathers had higher education levels tended to have higher average scores concerning career aspirations than those whose fathers had a lower educational level.

These results are in close agreement with the findings that career aspirations are affected by parental education and socioeconomic status, encompassing the opportunity, encouragement, and support that parents provide to children [11, 21–23], in terms of their aspirations and career development. This can be explained by the fact that Thai fathers or parents with a lower education level may not expend enough effort or energy in motivating their children to develop career aspirations. Moreover, a parent who has a lower level of education may not understand very much about child development at each moment in a child’s life, and this may be the reason for poor speech and listening skills displayed by parents when talking with their children [74], particularly regarding the skills or abilities that the child needs for adequate development. In the context of the Thai family, Thai adolescents are heavily influenced by their parents in their thinking or planning toward career and achievement [75].

The findings also support the hypothesis that self-esteem has an indirect effect on career aspirations through resilience and future orientation, although the total effect was relatively small. This suggests that self-esteem can help students develop their resilience and ability which will contribute toward their having a good future orientation which, in turn, leads to the development of a higher level of career aspiration. The findings confirm that self-esteem is a stable predictor of an individual’s behavior [76], which is in line with several previous studies concerned with the role and importance of self-esteem in human and career development [77–79]. Self-esteem is an attribute which increases the power of a person’s self or beliefs, and may be associated with an individual’s ability to achieve their goals and be successful in their career [80–82].

Since paternal education level is recognized as being an important factor in predicting career aspirations, the findings from this research suggest that Thai students, especially those who come from deprived backgrounds and educational limitations, are expected to proceed to an improved social and economic outcome. This can be achieved through the process of the socialization of the individual to lead them to achieve success in a career [83]. This would thus help university students discover their career aspirations. Such findings may be explained by the fact that according to Thai culture, parents and teachers advise students to succeed in a career, and tell them how important it is to receive academic qualifications that will potentiate socioeconomic status throughout their career. Therefore, resilience may be more challenging for Thai university students and enable them to build a future orientation aspect. University career counselors should work together with lecturers and other practitioners to increase students’ resilience. By using this approach, counselors may provide the knowledge and skills to support lecturers in building students’ strengths and improving their resilience during the pedagogical process in the classroom, a task which does not necessarily have to mean extra work in addition to teaching [84]. This method is the basis of a pedagogy that will gain effective results with students. Usually, in the Thai university context, lecturers and counselors are very influential in being able to help improve the mental health of students. Consequently, they can help a student develop resilience through offering workshops as well [85].

Furthermore, since the university environment has relationship with the students’ concepts and learning, universities should have a policy of cultivating career aspirations for students to improve their achievements, clarify their personal and career goals, as well as striving to develop high-level skills in their profession. Moreover, counselors, lecturers, and parents must help students discover their passions and career aspirations, as well as develop their competence, curiosity, self-esteem, resilience, and future orientation to achieve their career goals, including long-term career development.

### Limitations and suggestions for future research

Some limitations and suggestions for future research should be mentioned. Firstly, because one of the main objectives of this study was to develop and validate the proposed model for predicting career aspirations, a cross-sectional correlation study was chosen by design, in which causal relationship cannot be directly explained due to the inability to manipulate the independent variables. Therefore, future studies should take an experimental method to clearly understand the causal relationships among the variables in the model.

Secondly, this model was a starting point for studying the effects of paternal education and three affective variables for the career aspirations of Thai university students. Many more variables are likely to have an effect on their career aspirations, for example, the socioeconomic status, participant gender [86, 87], academic ability [11], classroom environments [88], reference groups [89], the quality of counseling [90], or the trickle-down effect [91]; the socioeconomic status was not directly taken into account in this study, because the survey was conducted in classrooms, in which not all students know instantly and exactly, for example, the income and occupational prestige of their family. Further research may consider exploring the association between these factors and career aspirations in the Thai context.

Thirdly, since the findings were based on a limited sample of undergraduate students from two universities in Thailand, a study encompassing multiple universities may help in increasing the validity of the results. Consequently, future studies should examine the generalizability of the results with more diverse university student samples, in which investigations should be undertaken into whether the practice of counselors, support staff, and faculty members has any impact on career aspirations.

Furthermore, the investigation of the association between career aspirations and factors in developing countries may be different from what is expected in developed countries. Therefore, further research should compare the invariance of the model between developing countries and developed countries as well as consider including student backgrounds (i.e., origin of the family and other socioeconomic factors; rural vs urban origins) and other factors in the model.

Finally, since this study focused only on public universities, further research should include participants from private universities or other groups, e.g., groups based on vocational education or age, and consider testing invariance of this model across different cultural groups and various disciplines.

Despite its limitations, this study indicates the relationships between career aspirations and paternal education, self-esteem, resilience, and future orientation of Thai university students. It also supports the continued use of the structural equation model in empirical research on career development.

## Supporting information

S1 Questionnaires The English and Thai version of career aspirations, future orientation, resilience, and self-esteem scales. (DOCX)

S1 Dataset (XLSX)
