# Supplementary material for: The relationship between paternal education, self-esteem, resilience, future orientation, and career aspirations
Source: PLoS One. 2020 Dec 8;15(12):e0243283. doi: 10.1371/journal.pone.0243283 (PMC7723283; doi:10.1371/journal.pone.0243283)
Supplement: S1 Questionnaires — (DOCX) [file pone.0243283.s001.docx]

**S1 Questionnaires.**

**The English and Thai version of career aspirations, future orientation, resilience, and self-esteem scales.**

**1) Career Aspiration Scale** used with permission from Dr. Karen M. O'Brien.

Source: http://counselingpsychologyresearch.weebly.com/career-aspiration-scale---revised.html

| **Factor** | **English Version** | **Thai Version** |
| --- | --- | --- |
| Leadership | 1. I hope to become a leader in my career field. | 1. ฉันหวังจะได้เป็นผู้นำในสาขาอาชีพของฉัน |
| Leadership | 2. I do not plan to devote energy to getting promoted to a leadership position in the organization or business in which I am working. (Reverse score) | 2. ฉันไม่มีแผนที่จะทุ่มเทพลัง เพื่อให้ได้เลื่อนตำแหน่งเป็นผู้นำในองค์กรหรือธุรกิจที่ฉันกำลังทำงานอยู่ (Reverse score) |
| Achievement | 3. I want to be among the very best in my field. | 3. ฉันต้องการอยู่ในกลุ่มที่ดีที่สุดในสายงานของฉัน |
| Leadership | 4. Becoming a leader in my job is not at all important to me. (Reverse score) | 4. การได้เป็นผู้นำในงานของฉัน ไม่สำคัญสำหรับฉันเลย (Reverse score) |
| Leadership | 5. When I am established in my career, I would like to manage other employees. | 5. เมื่อฉันมีความมั่นคงในอาชีพของฉัน ฉันอยากจะบริหารจัดการพนักงานคนอื่น ๆ |
| Educational | 6. I plan to reach the highest level of education in my field. | 6. ฉันมีแผนที่จะไปถึงระดับสูงสุดด้านการศึกษาในสาขาของฉัน |
| Leadership | 7. I want to have responsibility for the future direction of my organization or business. | 7. ฉันปรารถนาที่จะรับผิดชอบต่อทิศทางในอนาคตขององค์กรหรือธุรกิจของฉัน |
| Achievement | 8. I want my work to have a lasting impact on my field. | 8. ฉันต้องการที่จะให้ผลงานของฉัน มีผลกระทบต่อสาขาอาชีพของฉันในระยะยาว |
| Achievement | 9. I aspire to have my contributions at work recognized by my employer. | 9. ฉันปรารถนาที่จะมีส่วนร่วมในการทำงานที่นายจ้างของฉันรับรู้ |
| Educational | 10. I will pursue additional training in my occupational area of interest. | 10. ฉันจะหมั่นฝึกอบรมเพิ่มเติมในสายอาชีพที่ฉันสนใจ |
| Educational | 11. I will always be knowledgeable about recent advances in my field. | 11. ฉันมีความรู้ที่เกี่ยวข้องกับความก้าวหน้าล่าสุดในสายงานของฉันเสมอ |
| Leadership | 12. Attaining leadership status in my career is not that important to me. (Reverse score) | 12. การได้สถานะผู้นำในอาชีพของฉัน ไม่ใช่เรื่องสำคัญสำหรับฉัน (Reverse score) |
| Achievement | 13. Being outstanding at what I do at work is very important to me. | 13. การมีความโดดเด่นในสิ่งที่ฉันทำในงาน เป็นสิ่งสำคัญสำหรับฉัน |
| Educational | 14. I know I will work to remain current regarding knowledge in my field. | 14. ฉันรู้ว่าฉันต้องทำงานเพื่อธำรงไว้ซึ่งความรู้ในสาขาของฉัน |
| Leadership | 15. I hope to move up to a leadership position in my organization or business. | 15. ฉันหวังจะก้าวขึ้นในตำแหน่งผู้นำในองค์กรหรือธุรกิจของฉัน |
| Educational | 16. I will attend conferences annually to advance my knowledge. | 16. ฉันจะเข้าร่วมการประชุมเป็นประจำทุกปีเพื่อพัฒนาความรู้ของฉัน |
| Achievement | 17. I know that I will be recognized for my accomplishments in my field | 17. ฉันรู้ว่าฉันจะได้รับการยอมรับในความสำเร็จในสายงานของฉัน |
| Educational | 18. Even if not required, I would take continuing education courses to become more knowledgeable. | 18. ไม่ว่าจะจำเป็นหรือไม่ ฉันจะเข้าเรียนในหลักสูตรการศึกษาต่อเนื่องเพื่อให้มีความรู้มากขึ้น |
| Educational | 19. I would pursue an advanced education program to gain specialized knowledge in my field. | 19. ฉันจะศึกษาในหลักสูตรการศึกษาขั้นสูง เพื่อเพิ่มพูนความรู้เฉพาะทางในสาขาของฉัน |
| Achievement | 20. Achieving in my career is not at all important to me. (Reverse score) | 20. ความสำเร็จในอาชีพของฉัน ไม่สำคัญสำหรับฉันเลย (Reverse score) |
| Achievement | 21. I plan to obtain many promotions in my organization or business. | 21. ฉันวางแผนที่จะได้เลื่อนตำแหน่งต่าง ๆ ในองค์กรหรือธุรกิจของฉัน |
| Achievement | 22. Being one of the best in my field is not important to me. (Reverse score) | 22. การเป็นหนึ่งในบุคลากรที่ดีที่สุดในสายงานของฉัน ไม่ใช่สิ่งสำคัญสำหรับฉัน (Reverse score) |
| Educational | 23. Every year, I will prioritize involvement in continuing education to advance my career. | 23. ทุก ๆ ปี ฉันจะให้ความสำคัญกับการมีส่วนร่วมในการศึกษาต่อเนื่องเพื่อพัฒนาอาชีพของฉัน |
| Leadership | 24. I plan to rise to the top leadership position of my organization or business. | 24. ฉันวางแผนที่จะก้าวขึ้นสู่ตำแหน่งผู้นำสูงสุดขององค์กรหรือธุรกิจของฉัน |

**2) Future Orientation and Resilience** used with permission from Dr Ilaria Di Maggio.

Source: Di Maggio I, Ginevra MC, Nota L, Soresi S. Development and validation of an instrument to assess future orientation and resilience in adolescence. Journal of Adolescence. 2016;51:114-22. doi: 10.1016/j.adolescence.2016.06.005.

| **Factor** | **English Version** | **Thai Version** |
| --- | --- | --- |
| Future Orientation | 1. Looking ahead makes me feel full of energy. | 1. การมองไปข้างหน้า ทำให้ฉันรู้สึกเต็มเปี่ยมไปด้วยพลัง |
| Future Orientation | 2. I like thinking about where I'll find myself in a few years. | 2. ฉันชอบคิดว่า ฉันจะเป็นอะไรในอนาคตข้างหน้า |
| Future Orientation | 3. I have many aspirations for my future. | 3. ฉันมีแรงบันดาลใจมากมายสำหรับอนาคตของฉัน |
| Future Orientation | 4. I'm already passionate about the idea of realizing my dreams in the future. | 4. ฉันหลงใหลในความคิดในการทำให้ความฝันของฉันเป็นจริงในอนาคต |
| Future Orientation | 5. Thinking about the future excites me. | 5. การคิดเกี่ยวกับอนาคตทำให้ฉันตื่นเต้น |
| Future Orientation | 6. Thinking of my future life fills me with hope. | 6. การคิดถึงชีวิตในอนาคตของฉัน ทำให้ฉันเต็มไปด้วยความหวัง |
| Future Orientation | 7. I like daydreaming what future will reserve me. | 7. ฉันชอบการฝันกลางวันว่า อะไรในอนาคตจะเหมาะกับฉัน |
| Future Orientation | 8. I often think about building positive future for me. | 8. ฉันมักจะคิดถึงการสร้างอนาคตในเชิงบวกให้กับตน |
| Future Orientation | 9. I like to think about future goals I have still to carefully consider. | 9. ฉันชอบคิดถึงเป้าหมายในอนาคต ซึ่งฉันยังคงต้องพิจารณาอย่างรอบคอบ |
| Future Orientation | 10. I have some specific goals for my future. | 10. ฉันมีเป้าหมายชัดเจนสำหรับอนาคตของฉัน |
| Future Orientation | 11. When I think about my future I focus my attention on the type of person I want to become. | 11. เมื่อฉันคิดถึงอนาคต ฉันมุ่งความสนใจไปที่ประเภทของคนที่ฉันอยากจะเป็น |
| Resilience | 12. I consider myself a strong person | 12. ฉันคิดว่าฉันเป็นคนที่เข้มแข็ง |
| Resilience | 13. I always commit myself a lot to reach my goals. | 13. ฉันมักจะทุ่มเท เพื่อบรรลุเป้าหมายของฉัน |
| Resilience | 14. I consider myself able to tackle everything that may happen. | 14. ฉันคิดว่าตัวเองสามารถจัดการทุกอย่างที่อาจเกิดขึ้นได้ |
| Resilience | 15. Even under pressure, I'm able to concentrate, to think with finish and carefully. | 15. แม้ภายใต้ความกดดัน ฉันก็สามารถที่จะมีสมาธิ คิด ด้วยความรอบคอบ |
| Resilience | 16. I can see the ironic aspects of things | 16. ฉันสามารถมองเห็นแง่มุมที่ขบขันในสิ่งต่าง ๆ ได้ |
| Resilience | 17. I can handle it to achieve my goals | 17. ฉันสามารถรับมือเพื่อให้บรรลุเป้าหมายของฉันได้ |
| Resilience | 18. Dealing with stressful situations made me stronger | 18. การจัดการกับสถานการณ์ที่ตึงเครียด ทำให้ฉันเข้มแข็งขึ้น |
| Resilience | 19. I do not discourage a lot easily after a failure | 19. ฉันไม่ท้อง่าย ภายหลังจากความล้มเหลว |
